# Supplementary material for: Sb Nanoparticles Embedded in the N-Doped Carbon Fibers as Binder-Free Anode for Flexible Li-Ion Batteries
Source: Nanomaterials (Basel). 2022 Sep 6;12(18):3093. doi: 10.3390/nano12183093 (PMC9506069; doi:10.3390/nano12183093)
Supplement: Supplementary file 1 [file nanomaterials-12-03093-s001.zip › nanomaterials-1886447-supplementary materials.pdf]

nanomaterials

MDPI

Supplementary Materials

Sb Nanoparticles Embedded in the N-Doped Carbon Fibers as Binder-Free Anode for Flexible Li-Ion Batteries

Xin Wang [1], Nanjun Jia [1], Jianwei Li [1,*], Pengbo Liu [1], Xinsheng Zhao [1], Yuxiao Lin [1], Changqing Sun [2] and Wei Qin [3,*]

[1] School of Physics and Electronic Engineering, Jiangsu Normal University, Xuzhou 221116, China
[2] Research Institute of Interdisciplinary Science and School of Materials Science and Engineering, Dongguan University of Technology, Dongguan 523820, China
[3] College of Materials Science and Engineering, Changsha University of Science and Technology, Changsha 410114, China
* Correspondence: jwl189@163.com (J.L.); qinwei@csust.edu.cn (W.Q.)

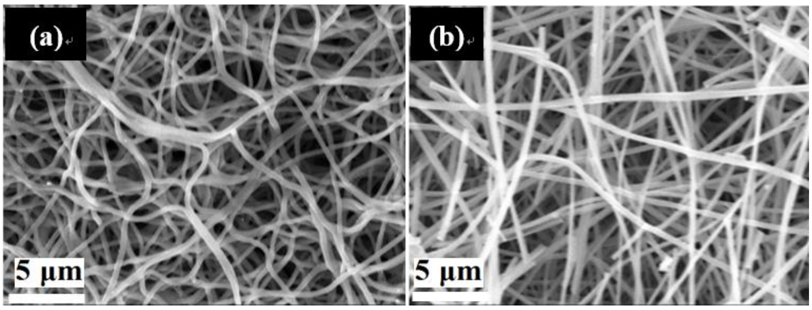

Figure S1. SEM images of (**a**) Sb_1 and (**b**) Sb_2.

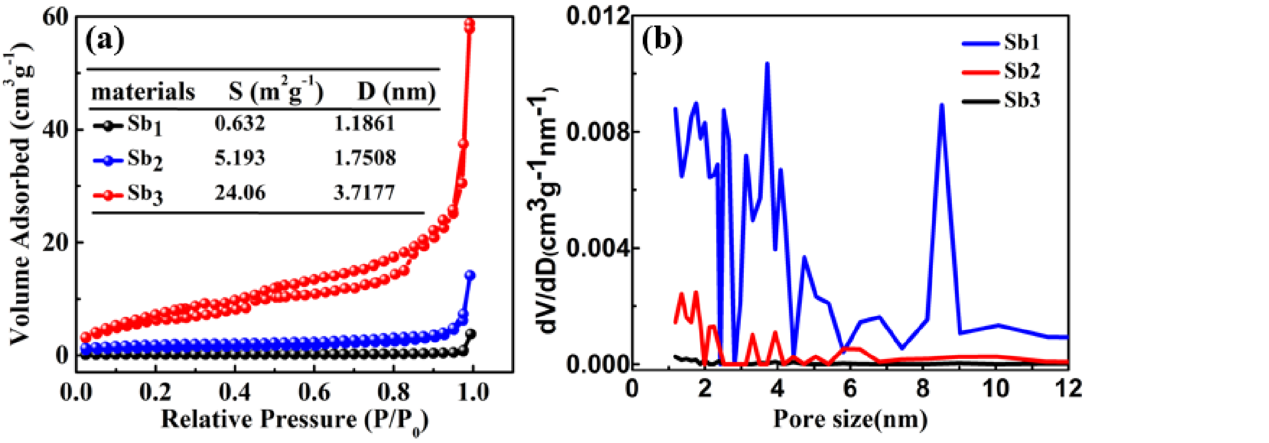

Figure S2. (**a**) N_2 adsorption-desorption isotherms and their as-calculated structural parameters in insert. (**b**) The pore size distribution of Sb_1, Sb_2, Sb_3.

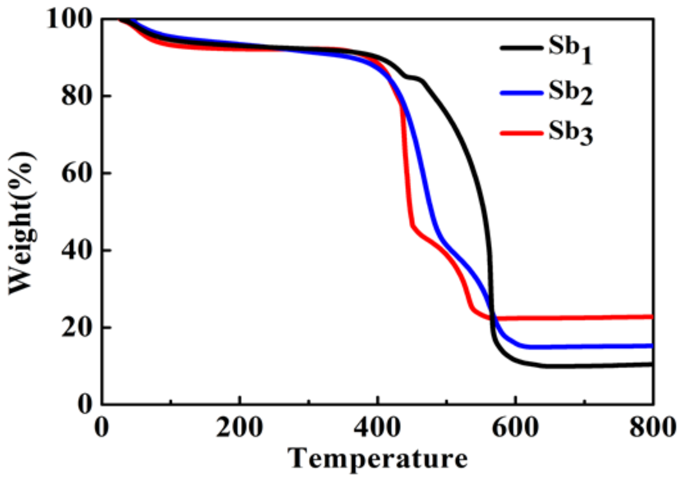

Figure S3. TGA curves of Sb_1, Sb_2 and Sb_3.
